# Effect of malaria prevention education on bed net utilization, incidence of malaria and treatment seeking among school-aged children in Southern Ethiopia; cluster randomized controlled trial

**DOI:** 10.1186/s12879-023-08464-w

**Published:** 2023-07-20

**Authors:** Zerihun Zerdo, Hilde Bastiaens, Sibyl Anthierens, Fekadu Massebo, Matewos Masne, Gelila Biresaw, Misgun Shewangizaw, Abayneh Tunje, Yilma Chisha, Tsegaye Yohannes, Jean-Pierre Van Geertruyden

**Affiliations:** 1 Department of Medical Laboratory Science, College of Medicine and Health Sciences, Arba Minch University, Arba Minch, Ethiopia; 2 Global Health Institute, Antwerp University, Antwerp, Belgium; 3 Department of family medicine and population health, Antwerp University, Antwerp, Belgium; 4 Department of Biology, College of Natural Sciences, Arba Minch University, Arba Minch, Ethiopia; 5 Department of Public Health, College of Medicine and Health Sciences, Arba Minch University, Arba Minch, Ethiopia

**Keywords:** Malaria prevention education, Bed net utilization, Incidence, Treatment seeking, School-aged children, Ethiopia

## Abstract

**Background:**

School-aged children (SAC) have an increased risk to contract malaria and play a major role in its transmission dynamics. However, their malaria prevention experience is poor. Thus, the effect of malaria prevention education (MPE) on bed net utilization, treatment seeking from a health facility and cumulative incidence of malaria was evaluated in Southern Ethiopia.

**Methods:**

A two arm cluster randomized controlled trial was conducted by recruiting 2038 SAC from 32 schools. Structured questionnaire was used to collect data on socio-demographic, economic, bed net ownership, bed net utilization, whether the participated child suffered from malaria and has got treatment from a health facility. Generalized mixed effect logistic regression using school as random variable was used to assess the effect of the intervention on the outcome variables.

**Results:**

The ownership of bed net in households of the control and intervention schools was similar respectively with 84.6 and 88.6% (Crude Odds Ratio (COR): 1.5; 95%CI: 0.5–4.8). The percentage of SAC slept under the bed net the night before the survey was also similar (55.1% versus 54.0%); COR:1.04; 95%CI: 0.5–2.4). Bed net utilization was affected by household size to the bed net ratio ≤ 2 (Adjusted Odds Ratio (AOR) = 1.6; 95%CI:1.3–2.1), bed net utilization at baseline of the study (AOR = 2.3; 95%CI:1.5–3.6), and history of malaria attack in the last twelve months (AOR = 1.3; 95%CI:1.01–1.8). Reported cumulative incidence of malaria and treatment seeking from a health facility by SAC was similar between intervention and control arms: -2.1% (COR = 0.8; 95%CI: 0.5–1.5) and 9.6% (COR = 1.4; 95%CI: 0.4–4.3) respectively. The reported incidence of malaria was affected by altitude (AOR = 0.5; 95%CI: 0.3–0.8), low and medium wealth index (AOR = 0.7; 95%CI: 0.5–0.96 and AOR = 0.7; 95%CI: 0.5–0.98), adequate bed net number for household members (AOR = 0.7; 95%CI:0.5–0.9) and bed net utilization (AOR = 1.3; 95%CI:1.1–1.8).

**Conclusions:**

MPE had no significant effect on the use of malaria prevention measures considered, treatment seeking from a health facility and reported cumulative incidence of malaria though bed net use was associated with malaria incidence. Before organizing any health education program, sustainable implementation efforts have to be warranted especially in SAC, a neglected but relevant vulnerable and reservoirs.

**Trial registration:**

Pan African Clinical Trials Registry PACTR202001837195738, registered 21/01/2020.

## Introduction

Malaria remains the leading cause of morbidity and mortality in economically disadvantaged tropical and subtropical regions, particularly in Africa [1–3]. Of the total 241 million malaria cases and 627 thousand deaths due to malaria, 95% of the cases and 96% of the deaths occurred in the WHO African region in the year 2020 [1]. Due to widespread efforts and intensified use of malaria prevention measures, the burden of malaria declined in the last two decades [2, 4, 5]. Vector control measures such as long-lasting insecticidal bed nets (LLINs) and indoor residual spray (IRS) and prompt diagnosis and treatment of malaria by using artemisinin based combination therapies (ACTs) are the major malaria prevention measures used. Between 2004 and 2020, bed net manufactures distributed more than 2.3 billion bed nets. The proportion bed net utilization among children aged below five years increased from 3% in 2000 to 49% in 2020 [1]. LLINs was accountable for 68% of 663 million clinical cases of malaria averted between 2000 and 2015 [6]. Recent evidences suggest that LLINs use in early life has an impact on reducing mortality in adulthoods [7].

As the transmission intensity reduces due to the widespread use of malaria prevention measures, there is change in age pattern of malaria. The proportion of clinical malaria among under-five children was above 60% in high endemic settings but the same age group account for below 20% in low transmission settings [8]. The decline in malaria transmission leads to less exposure of children under the age of five years to malaria parasites; thus, delaying the specific acquired malaria immunity in school-aged children (5–14 years) (SAC). As a result of delayed development of functional antimalaria immunity and low benefit from the malaria prevention measures, SAC become at increased risk of malaria and associated morbidities [9–13]. A hospital based retrospective cohort study conducted in Mozambique also indicated patterns of patients with malaria and malaria deaths shifted towards older ages over the study period. The percentage of patients with malaria occurring among children aged 5 years or older increased from 8 to 13% in 1997–2006 to 26–33% in 2013–2017 [14]. Nearly half of SAC in sub-Saharan Africa (SSA) were infected by plasmodium species. The prevalence of Plasmodium infection was 74.8% in Cameron [15], 73.9% in Ivory Coast [16], 5.4–57.5% in Tanzania [17–19], 31% in Malawi [20], and 22.4% in Rwanda [21].

Most of these infections among SAC are asymptomatic and not presented to a health facility for treatment to halt either progression to clinical or severe malaria and/or interrupt disease transmission [20, 22]. These infections persist for a long duration than the infections in their under-five counterparts as indicated by a study in Malawi [9]. They also carry many gametocytes responsible for disease transmission. Therefore, SAC are a major malaria reservoir threatening malaria control and elimination efforts [23–25]. From the infected SAC perspective, asymptomatic infections are converted to clinical malaria in later times [26, 27]. The early conversion of such infections to clinical malaria was affected by anemia and the female gender in Cameroon [28]. Malaria is still responsible for 50% of age-specific mortality among SAC in SSA [22].

Unfortunately, SAC are the least likely to get proven malaria intervention measures such as LLINs and prompt diagnosis and effective treatment. Their care givers visit informal shops for the management of their clinical malaria and are less likely to sleep under insecticide-treated bed nets as compared to other population segments [9, 13, 20, 29]. In contrast to the high prevalence of malaria, only 43% of SAC slept the previous night under a bed net as revealed by countrywide survey conducted in Ivory Coast in the late 2012 [16]. After national bed net distribution in 2017, bed net use among children aged 5–15 years in Uganda was 30.7%, which is lower than the bed net use among children under the age of five years (44.7%) and inhabitants over the age of 15 years (44.1%) [13, 16].

Ethiopia is one of the malaria-endemic countries in the SSA, and both *P. falciparum* and *P. vivax* are prevalent. About 52% of the total population in Ethiopia is at risk of malaria and the primary mosquito responsible for transmission is *Anopheles arabiensis* of the *Anopheles gambiae* complex, a highly efficient vector [29, 30]. From 2013 till the burst of COVID-19, the number of malaria cases and deaths consistently decreased over time [1, 2, 30]. According to the 2015 Ethiopian malaria indicator survey, the highest prevalence of malaria (1.5%) was observed in children of the age category 9–11 years [30]. However, there is enormous variability (i.e. in Oromia region, 14.5% of the children were positive [31] and in our study area the prevalence of malaria among SAC was 1.62% [32].

Prompt diagnosis and treatment of malaria and vector control measures are the major malaria prevention practices in Ethiopia. As a means of disease prevention, Ethiopia has deployed a huge workforce of health extension workers in the fight against malaria in 2004. At the same time, Ethiopia introduced LLINs (distributed free of charge) [30, 33]. LLINs are targeted to households living in all malaria endemic areas with the target of universal coverage (100%) of one LLIN for every two individuals in the household [34]. The national coverage of bed net utilization conditional to owning at least one bed net was 61% [31]. However, our estimate at baseline of this study was lower (40.4%) than the national coverage [35]. Concerning the treatment seeking behavior, about 37.3% of the residents suspected for malaria used some sort of self-intervention for the treatment of malaria as indicated from a survey undertaken in one of the kebeles, the lowest administrative unit, in Ethiopia. The self-interventions used were use of modern medications not prescribed by health professionals, using herbs and non-pharmacological interventions [36]. In Dangila town in North western Ethiopia, the prevalence of modern health care seeking was 82.1% and it is highly influenced by severity of malaria symptoms [37].

To overcome the underutilization of malaria prevention measures and broader strategies to control and eliminate malaria, different researches suggest the use of targeted and well-tailored awareness creation to increase the utilization of malaria prevention measures [29, 38–42]. A study from South Africa revealed that knowledge about the protective efficacy of bed net was responsible for a 30–40% increase in bed net utilization [41]. School-based interventions are highly efficient and effective as has been seen with the distribution of mass drug administration for SAC against soil-transmitted helminthiasis and schistosomiasis [43]. School-based malaria education, bed net distribution and intermittent parasite clearance medications given to SAC in the school compound in Malawi [44] drastically reduced malaria. In similar, malaria participatory education given to SAC in Ghana reduced the prevalence of malaria from 30.1 to 10.3% [45]. However, the information from these studies is not adequate for policy makers and such interventions could vary widely depending on the context of the study. Thus, this study aimed to evaluate the effect of malaria prevention education given to SAC and their parents on bed net utilization and ownership, reported cumulative incidence of malaria, and prompt diagnosis and treatment of malaria by using cluster randomized controlled trial. The cluster randomized trial was used to minimize information contamination from the intervention group to the control group.

## Methods

### Study area and participants

**Fig. 1:**
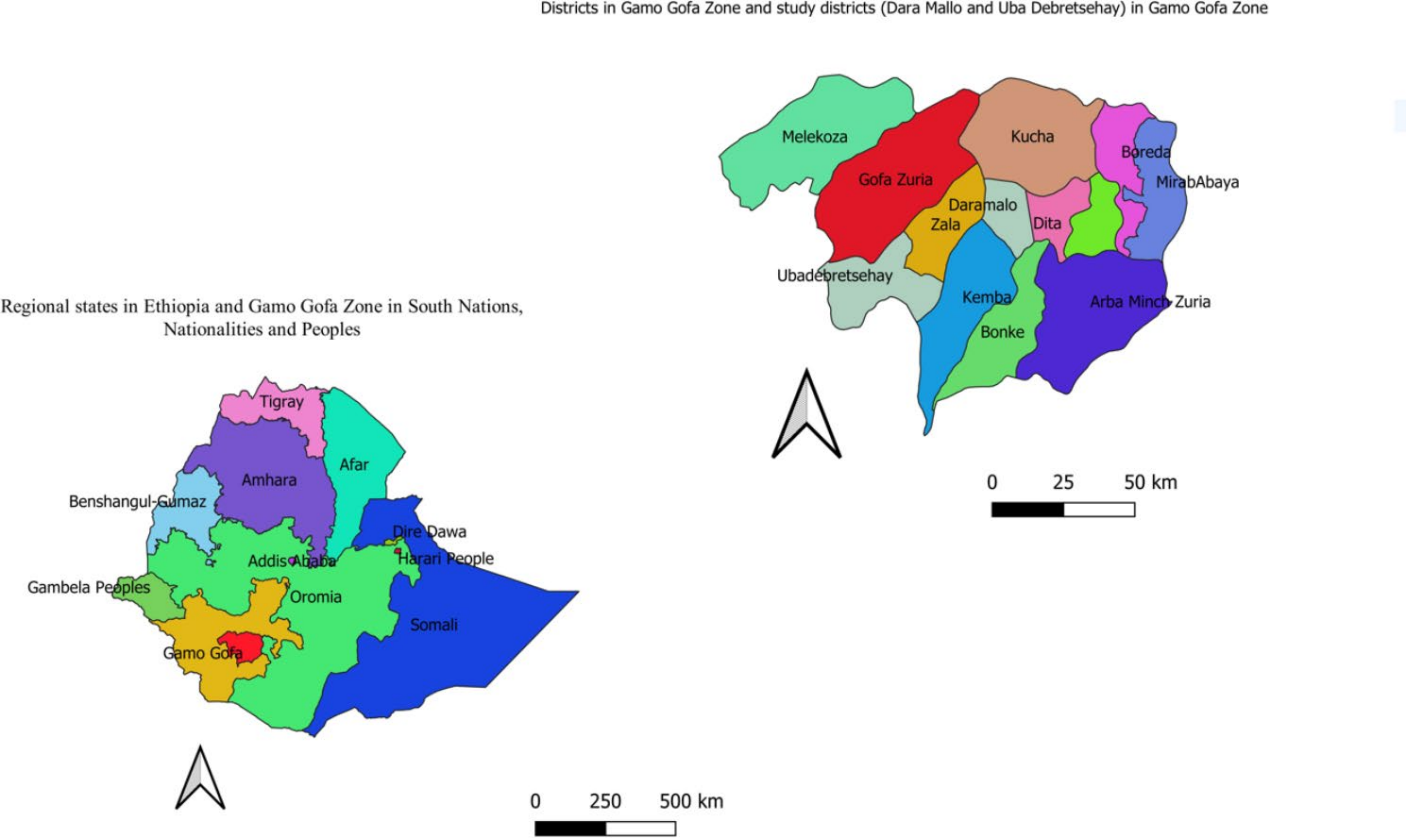
Study area in Gamo Gofa Zone in South Nations, Nationalities and peoples regional state, 2019

This study was conducted in Dara Mallo and Uba Debretsehay districts in Gamo and Gofa Zones respectively. These districts were selected as they harbor the highest burden of malaria in the former Gamo Gofa Zone in Southern Nations, Nationalities, and Peoples Regional (SNNPR) state. In line with the national malaria elimination program, the major malaria prevention measure in use in the two districts is LLINs. The study area was in the western part of Arba Minch town, the capital of the former Gamo Gofa zone (Fig. 1**)**. The population size based on the 2007 [35] national census and recent updates made by the respective districts was described in our previous article [46]. The recent update made by each district indicated that a total of total of 94,396 and 110,207 people were living in the Uba Debretsehay and Dara Mallo districts respectively. All 32 schools (20 from Uba Debretsehay and 12 from Dara Mallo) are in malaria transmission (at an altitude range below 2000 m above sea level MASL) parts of the districts were involved in the study.

### Trial design and participants

A 1:1 two-arm parallel open cluster randomized controlled trial was conducted to address the trial objectives. The trial was registered in Pan African Clinical Trials Registry (PACTR202001837195738). Clusters are primary schools that are found in malaria transmission settings in the two districts and enrolled at least 72 children aged 5–14 years. There were a total of 32 eligible primary schools in the two districts. Half of these schools were allocated to the intervention and the other half in the control groups. From each of these schools, 72 children attending their education from grade one to three, mentally fit to respond questions directed to them and without any physical problem to measure their height were included in the study. The selected 72 children and one of their parents in the intervention schools and only the selected SAC from the control arm of the trial participated in the study. An equal number of children were approached to be enrolled in the study from each school. The cluster randomized trial was used to minimize the information contamination from the intervention schools to the people in the control schools.

### The intervention and its implementation

The intervention was malaria prevention education. The content of the intervention package was developed after exploring the parent’s perception of the cause of malaria and their malaria prevention experience [47] and literature review. The intervention was given to children and their parents separately. The methods used for the delivery of the intervention were lecture and demonstration. The intervention providers explored the perception of the participants about the cause of malaria, which highly influenced their malaria prevention experiences. Once their perception of cause of malaria is explored by the participants, the trainers provided the right cause of malaria as transmission occurs through the bite of female Anopheles mosquitoes. The consequence of malaria is also discussed with the participants: death, anaemia, impaired development of children, economic loss, psychological trauma if parents were unable to treat their child, school absenteeism and associated poor school performance. The other topic covered in the intervention was prevention of malaria. The benefit of LLINs as a major malaria prevention measure and the proper use of bed net is demonstrated. Here the misuse of the bed nets by different individuals which we have observed during our qualitative study were discussed [47]. The other malaria prevention measures discussed were the indoor residual spray and larval source management. The symptoms of malaria were also discussed and as a child should be taken to a health facility as soon as possible after the onset of malaria symptoms.

Two science teachers and a school director from each intervention schools were trained by the research team from Arba Minch University. The trained teachers provided the intervention to the selected SAC and their parents separately. The intervention was planned to be delivered twice per annum, once each semester. However, due to the COVID-19 pandemic and associated prevention measures, the intervention was given once in December 2019. To minimize dropouts and increase adherence to the intervention, sensitization of the students as there is malaria prevention education was done about two days before the date of delivery of the intervention by the trained teachers. Nothing is done to students in the control arm except prevention measures underway by the national malaria control program.

**Fig. 2:**
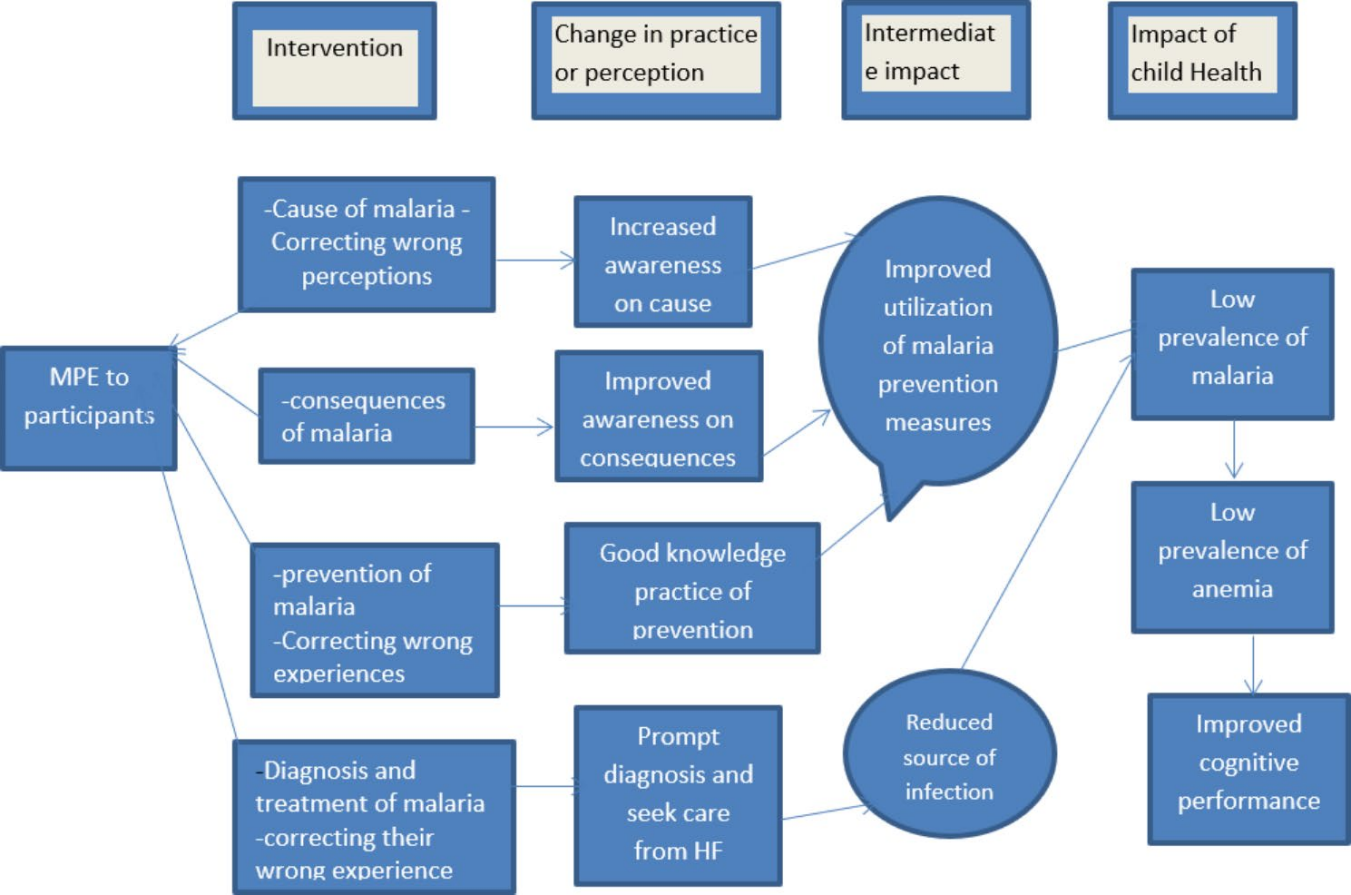
Model on causal pathway of MPE on malaria, anaemia and cognitive development of SAC, 2019

The intervention was hypothesized to reduce the prevalence of malaria and associated morbidities among SAC. As indicated in Fig. 2 the lecture given on the contents mentioned above and demonstration on how to properly fix bed nets would improve knowledge about the cause of malaria, consequence of malaria and malaria prevention measures. These in turn lead to improved use of malaria prevention measures and prompt diagnosis and effective treatment of malaria, which leads to a reduction in the source of infection. Reduction in the source of infection through prompt treatment of malaria and improved use of malaria prevention measures lead to a decreased malaria prevalence that in turn prevents malaria anaemia and prevention of anaemia leads to improved cognitive performance of SAC.

## Outcomes

The initial aim of the study was to assess the effect of MPE on incidence of malaria, anemia, and cognitive performance of SAC. However, because of the low prevalence of malaria than assumed, the outcomes of the intervention were changed to the proxy indicators of malaria. The primary and secondary outcomes used to evaluate the effectiveness of the intervention were described below.

### Primary outcome

The primary outcome of the trial was bed net utilization by SAC. It was defined as a percentage of SAC that slept under LLINs the night before the interview irrespective of bed net ownership.

### Secondary outcomes

The secondary outcomes of the trial were self-reported cumulative incidence of malaria, the percentage of children with symptoms of malaria who sought treatment from a health facility, and bed net ownership. Bed net ownership was defined as the presence of at least one bed net in the household. Reported cumulative incidence of malaria was defined as the percentage of SAC that self-reported suffering from malaria after the baseline data collection was completed. Percentage of SAC sought treatment from a health facility for malaria symptoms was defined as the percentage of SAC that sought treatment only from a health facility as the 1st choice to the total number of children reported as suffered from malaria during the follow-up period.

### Sample size

The bed net utilization among SAC in primary school was 30.7% in Uganda [13]. Knowledge about the protective effect of bed nets was demonstrated to increase bed net utilization by 30–40% in southern Africa [41]; thus, in this study we assumed a 30% increase in the bed net utilization by SAC. Other assumptions were a 0.025 intracluster correlation coefficient (ICC) and a 15% loss to follow-up or non-response rate. Based on the above assumptions, the estimated sample size was 729 children and 10 clusters per arm.

The estimated sample size for malaria and anemia as outcome variables was 16 clusters with the minimum cluster size of 72 and 1152 children in each of the intervention and control arms. To assess the effect of the intervention on the bed net utilization as primary endpoint and other secondary outcomes mentioned above, all children enrolled in the 16 clusters per group were included in the final analysis [48, 49].

### Sampling techniques and data collection

A total of 3,204 children attending their primary education were approached at 32 primary schools. Seventy-two children from grades one to three were selected using systematic random sampling technique from eligible children in each section of the students with a class roster as the sampling frame. The number of participants from each grade level (grade 1 to 3) was determined by their relative contribution to the total enrolment of students in the school. Children aged 5–14 years and attending their education in the schools during the data collection period were included in the study, but those mentally not fit to respond to questions directed to them and with physical problems to measure their height were excluded from the study. The selected children were used to trace their households. Participants’ households were approached by trained data collectors for interview.

A pretested, structured questionnaire was used to collect data on demographics, water source, toilet structure, household assets, and bed nets. The questions were adapted to the local context from the Demographic and Health Surveys (DHS) malaria indicator survey household questionnaire [50]. In addition, the presence or absence of bed net in the household, whether the participating child slept under the bed net the night before the survey, whether the child suffered from malaria after the baseline data collection was complete or not were assessed by using structured questionnaire. The questionnaire was uploaded to tablets in Open Data Kit (ODK) data collection application. The data collectors were trained on how to use the data collection tool and ethical procedures. The data collection occurred at two-time points: one at the baseline (October to December 2019) of the study and the other at the end (November and December 2020). In addition, the coordinates of the household location were collected using a global positioning system (GPS) during the baseline study. The data collectors interviewed mothers or caregivers (when the selected child’s mother was not available) and observed the toilet structure and the number and position of bed nets (hanged over sleeping place or not) in the households at the baseline of the study.

After baseline data were collected, the 32 schools were stratified into districts (Dara Mallo or Uba Debretsehay) and residence places (rural or urban). From each of these strata, schools were randomly assigned to intervention and control arms by a statistician who was not aware of schools. The final interview was made with either the parents or adults in the household. The data collectors were supervised daily for completeness and correctness of the data by supervisors from Arba Minch University.

### Data analysis

The data collection and analysis methods used were described elsewhere [32]. Specifically, the data collected by using the ODK data collection tool were converted into a comma-separated value (CSV) files by using the ODK briefcase. Multiple factor analysis by using household assets, housing conditions, source of drinking water, agricultural land area, and the number of domestic animals was used to generate the wealth index of a household. The 1st dimension was classified into tertiles to classify the household economic status into poor, medium, and high. An intention-to-treat analysis at an individual level was used to assess the effectiveness of the intervention. The primary outcome of the study is the difference in bed net utilization between SAC in the intervention arm and the control arms of the trial. The secondary outcomes are the differences in the reported cumulative incidence of malaria, seeking treatment for malaria from a health facility as the 1st choice and bed net ownership between the intervention and control arms. In addition, determinants of bed net utilization and reported cumulative incidence were the other secondary analysis included.

Univariable mixed effect logistic regression using glmer function of R version 4.0.4 statistical software was used to assess the effect of MPE on outcome variables by taking schools as a random variable to account for the cluster effect. Odds ratio (OR) and corresponding 95% confidence interval (CI) were used to assess the strength of association between the outcome variables and the intervention. For bed net utilization and reported incidence of malaria, whether there is a confounding effect or not is analyzed by using multivariable mixed-effect logistic regression. Therefore, apart from the intervention, other factors predicting the bed net utilization and reported cumulative incidence were assessed. The fit of the model in predicting the outcome variables was checked by Akaike Information Criterion (AIC). The selection of variables for multivariable mixed-effect logistic regression was made through the backward stepwise variable selection method in which the variable with the largest P-value is removed from the model and checked for AIC. If the removal of a variable from the model improves the AIC value, it is removed from the model, otherwise, it is retained in the final model. For multivariable mixed-effects models, P-values less than 0.05 were considered statistically significant.

## Results

**Fig. 3:**
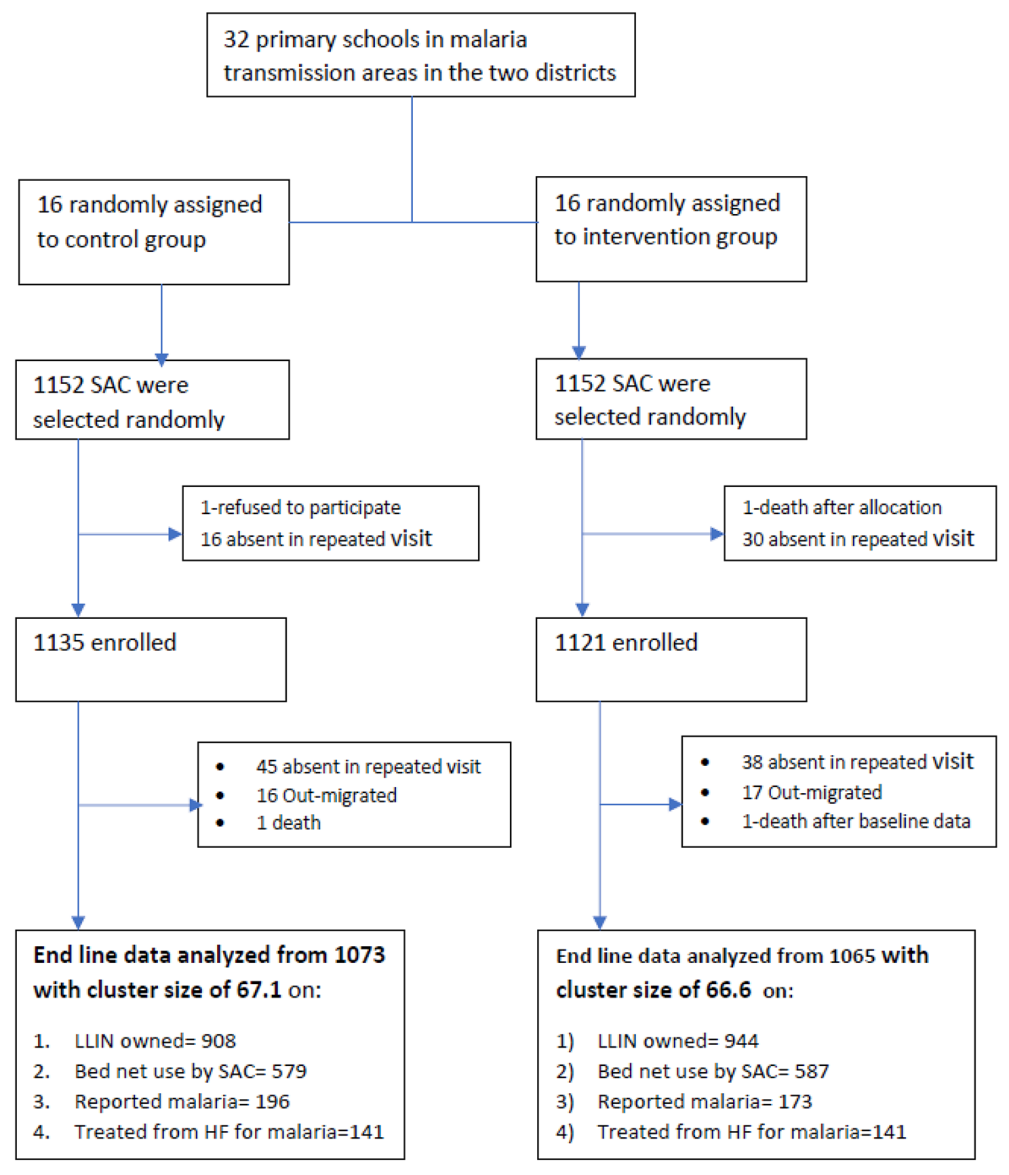
Flow diagram of clusters and participants in the MPE trial in Southern Ethiopia, 2019–2020

A total of 2304 SAC were approached for enrollment in the study. Of these, 2156 (93.6%) participants were consented and enrolled in the study. For evaluation of the intervention effectiveness, data from a total of 2138 (92.8% response rate) were analyzed and 118 were lost from the follow-up. Most common reasons for not enrolled in the study or lost from the follow-up were the mothers or caregivers being not available in repeated visits (two times), out-migration during the follow-up, refusal to participate due to underlying morbidity and death (Fig. 3**)**.

The mean number of SAC involved per cluster in the analysis, was 67.1 in the control arm and 66.6 in the intervention group. The background characteristics of SAC and their household sociodemographic and economic characteristics in both the intervention and control arms were depicted in Table 1. About 83.6% and 89.3% of participants were from rural areas in control and intervention group respectively. About 50.9% of children in the control group and 48.5% in the intervention arm were male. At baseline of the study, the bed net ownership was 19.2% in the control group while it was 18.2% in the intervention arm. The respective overall bed net utilization by SAC irrespective of bed net ownership were 7.0 and 7.9%. In most of the households, the bed nets owned were not adequate for the household size. The percentage of owning adequate number of bed nets for the household members in the control group was 10.7% while it was 7.9% in the intervention group.

**Table 1:** Baseline and sociodemographic characteristics of study participants in control and MPE intervention group of in Southern Ethiopia, 2019–2020

Variable	Categories	Control		Intervention	
		Number	Percent	number	Percent
District	Dara Mallo	395	36.8	401	37.7
	Uba Debretsehay	678	63.2	664	62.3
Residence place	Rural	897	83.6	951	89.3
	Semi/urban	176	16.4	114	10.7
Age of SAC	≤ 10	823	76.7	740	69.5
	> 10	250	23.3	325	30.5
Gender of the SAC	Female	527	49.1	548	51.5
	Male	546	50.9	517	48.5
Grade of SAC at enrollment	One	419	39.1	421	39.5
	Two	312	29.1	289	27.1
	Three	340	31.7	355	33.3
Occupational status of the household head	Civil servant	59	5.5	81	8.5
	Farmer	865	80.6	909	85.4
	Housewife	41	3.8	21	2.0
	Merchant	63	5.9	41	3.8
	Others	45	4.2	13	1.2
Educational status of SAC mother	Illiterate	776	72.3	783	73.5
	Literate	297	27.7	282	26.5
Education level of SAC mother	≤ grade 6	182	65.7	140	53.8
	>grade 6	95	34.3	120	46.2
Is there a pregnant woman in the household?	No	402	37.5	330	31.0
	Yes	671	62.5	735	69.0
Is there under 5 children in household?	No	402	37.5	330	31.0
	Yes	671	62.5	735	69.0
Baseline bed net ownership	Not owned	867	80.8	871	81.8
	Owned	206	19.2	194	18.2
Household size to bed net ratio	> 2 (inadequate)	184	89.3	175	90.2
	<= 2 (adequate)	22	10.7	19	9.8
Baseline bed net utilization of SAC	Not utilized	998	93.0	981	92.1
	Utilized	75	7.0	84	7.9
Baseline Plasmodium infection status	Not infected	995	98.2	1137	98.5
	Infected	18	1.8	17	1.5
Wealth index in tertiles	Low	377	35.1	353	31.5
	Medium	336	31.3	376	35.3
	High	360	33.6	376	33.1

### Effect of the intervention on outcome variables

The mean number of parents and SAC attended the MPE in each school were 62.9 and 93.5% respectively. The difference in bed net ownership before the intervention and after intervention in the control arm was 65.4% (84.6–19.2%) while it was 70.4% in the intervention group. The corresponding numbers for bed net utilization among SAC were 47.0 and 47.2 respectively. However, the differences in the bed net ownership as well as the difference in bed net utilization by SAC in the intervention and control group was not statistically significant (Table 2).

The reported number of malaria cases was higher in the control schools as compared to children in the intervention group. The reported cumulative incidence of malaria within one year of follow-up among children in the control group was 18.3%, but it was 16.2% in the intervention group. This difference was also not statistically significant (COR = 0.8; 95%CI of 0.5–1.5). The reported malaria cases sought treatment for malaria from different sources: health facilities (76.4%), taking antimalarial treatment from the private pharmacies without health professionals prescription (13.0%), traditional medicine (1.1%), homemade remedies (5.4%), and religious leaders (1.1%). The number of children who visited a health facility as the 1st choice after noticing the symptoms of malaria was 81.5% in the intervention arm and 71.9% in the control group. However, the difference (9.6%) was not statistically significant (COR = 1.4; 95%CI of 0.5–4.3). The detailed effect size of the intervention on the outcome variables was described in Table 2.

**Table 2:** Analysis of the effect of malaria prevention education on study outcomes and its effect size taking control groups as a reference, 2020

Outcome variable	# (%) yes	Study group		Difference	COR (95%CI)	P-value
		#(%) No **MPE**	#(%) **MPE**			
Household owned bed net	1852(86.6)	908(84.6)	944(88.6)	4.0%	1.5 (0.5–4.8)	0.48
Utilized bed net	1166(54.5)	579(54.0)	587(55.1)	1.1%	1.04 (0.5–2.4)	0.92
Reported that SAC suffered malaria	369(17.3)	196(18.3)	173(16.2)	-2.1%	0.8 (0.5–1.5)	0.57
Malaria suffered SAC visited a health facility	282(76.4)	141(71.9)	141(81.5)	9.6%	1.4 (0.5–4.3)	0.53

Apart from the intervention, other factors affecting the bed net utilization by the SAC were also assessed. In the univariable mixed effects logistic regression, several factors significantly affected bed net utilization by the SAC. Children from households where the household head is a civil servant were about 2 times more likely to slept under the bed net the previous night before the survey (95%CI of 1.2–3.2). The other factors significantly influenced bed net utilization by SAC were the educational status of the child’s mother/caretaker (COR = 1.3; 95%CI of 1.1–1.7), mothers/caretakers who were educated more than grade six (COR = 1.3; 95%CI of 1.3–1.7) as compared to those who did not reach to grade six among literates ones. Table 3 shows univariable analyses of factors affecting bed net utilization by SAC while Table 4 shows its multivariable analysis. The bed net utilization of SAC at baseline of the survey (AOR = 2.3; 95%CI of 1.5–3.6), the household size to the bed net ratio less than or equal to 2 (COR = 1.6; 95%CI of 1.3–2.1) and reported malaria (COR = 1.3; 95%CI of 1.01–1.8) were significantly associated with the bed net utilization in both univariable and multivariable analysis.

**Table 3:** Univariate analysis of bed net utilization by SAC in a MPE intervention study Southern Ethiopia, 2019–2020

Variable	Categories	#(%)SAC slept under bed net	COR (95%CI)	P-value
Intervention group	No MPE	579(54.0)	Ref	0.92
	MPE	587(55.1)	1.04(0.5–2.3)	
Residence place	Rural	982(53.1)	Ref	0.33
	Semi/urban	184(63.4)	1.53(0.7–3.6)	
Age of SAC	≤ 10	878(56.1)	Ref	0.10
	> 10	288(50.4)	0.9(0.8–1.01)	
Gender of the SAC	Female	591(55.0)	Ref	0.69
	Male	575(54.1)	1.0(0.8–1.2)	
Occupational status of the household head*	Farmer	939(52.9)	Ref	
	Civil servant	101(72.1)	2.0(1.2–3.2)	0.01
	Housewife	34(54.8)	1.1(0.6-2.0)	0.74
	Merchant	63(60.6)	1.5(0.9–2.4)	0.12
	Others	29(50.0)	1.2(0.7–2.2)	0.52
Educational status of SAC mother/caretaker*				
	Illiterate	809(51.9)	Ref	0.02
	Literate	357(61.7)	1.3(1.1–1.7)	
The education level of SAC mother is conditional on she is literate*				
	≤ grade 6	189(58.7)	Ref	0.02
	>grade 6	147(68.4)	1.3(1.1–1.7)	
Is there a pregnant woman in the household of the study SAC?				
	No	1038(54.8)	Ref	0.58
	Yes	128(52.7)	0.9(0.7–1.2)	
Is there an under 5 years old child in the household?				
	No	420(57.2)	Ref	0.52
	Yes	746(53.1)	0.9(0.8–1.2)	
owned bed net at baseline*	No	908(52.2)	Ref	< 0.01
	Yes	258(64.5)	1.7(1.3–2.3)	
Household size to bed net ratio conditional to owning adequate bed net to the household size**				
	> 2(no)	821(60.2)	Ref	< 0.01
	≤2(yes)	340(71.0)	1.6(1.3–2.1)	
Is the study child passed under the bed net at the bassline survey? **				
	Not utilized	1054(53.3)	Ref	< 0.01
	Utilized	112(70.4)	2.4(1.6–3.7)	
Wealth index of the household in tertiles				
	Low	377(52.9)	0.9(0.7–1.1)	0.24
	Medium	375(52.7)	1.0(0.8–1.2)	0.82
	High	414(58.1)	Ref	
	No	934(52.8)	Ref	0.04
	Yes	232(62.9)	1.3(1.02–1.7)	
The altitude of the residence above MASL	≤ 1100	306(39.2)	Ref	
	1100–1250	419(58.7)	0.8(0.6–1.2)	0.32
	> 1250	339(52.7)	0.9(0.6–1.6)	0.81

*Statistically significant at univariable analysis

**Table 4:** Predictors of bed net utilization by SAC in a MPE intervention study Southern Ethiopia, 2019–2020

Variable	Categories	#(%) SAC used bed net	AOR(95%CI)	P-value
Intervention group	No MPE	579(54.0)	Ref	
	MPE	587(55.1)	1.0 (0.5–1.8)	0.84
Household size to bed net ratio conditional to owning the bed net is adequate for the household size**				
	> 2(no)	821(60.2)		< 0.01
	≤2(yes)	340(71.0)	1.6(1.3–2.1)	
Is the study child passed under the bed net at the bassline survey? **				
	Not utilized	1054(53.3)	Ref	< 0.01
	Utilized	112(70.4)	2.3(1.5–3.6)	
Reported that the child suffered from malaria during the follow-up**				
	No	934(52.8)	Ref	0.04
	Yes	232(62.9)	1.3(1.01–1.8)	

** statistically significant at multivariable analysis

Tables 5 and 6 show univariate and multivariable mixed effects logistic regression of factors affecting reported incidence of malaria among SAC. In multivariable mixed effects logistic regression, the reported cumulative incidence of malaria was associated with altitude of the residence house, wealth index of the household, adequate access to the bed net and SAC slept under the bed net the night before the end line survey (Table 6**)**. Children residing at an altitude above 1250 MASL (AOR = 0.5; 95% CI: 0.3–0.8), adequate number of bed nets for the household members (AOR = 0.7; 95% CI: 0.5–0.9), a child slept under the bed net the night before the end line survey (AOR = 1.4; 95% CI: 1.1–1.8), those from the low (AOR = 0.7; 95% CI: 0.5–0.96), and medium (AOR = 0.7; 95% CI: 0.5–0.98) wealth index as compared to others in the high wealth index tertiles were factors associated with reported cumulative incidence of malaria.

**Table 5:** Univariate analysis of reported malaria incidence among SAC in a MPE intervention study in Southern Ethiopia, 2019–2020

Variable	Categories		COR (95%CI)	P-value
		#(%) of reported malaria		
Intervention group	No MPE	196 (53.1)	Ref	0.57
	MPE	173 (46.9)	0.8 (0.5–1.5)	
Residence place	Rural	303 (82.1)	Ref	0.57
	Semi/urban	66 (17.9)	1.3 (0.6–2.6)	
Age of SAC	≤ 10	266 (72.1)	Ref	0.46
	> 10	103 (27.9)	1.1 (0.8–1.5)	
Gender of the SAC	Female	179 (48.5)	Ref	0.52
	Male	190 (51.5)	1.1 (0.9–1.4)	
Occupational status of the household head	Farmer	300 (81.3)	Ref	
	Civil servant	36 (9.8)	1.4 (0.9–2.4)	0.17
	Housewife	6 (1.6)	0.5 (0.2–1.2)	0.14
	Merchant	19 (5.1)	0.9 (0.5–1.6)	0.75
	Others	8 (2.2)	0.6 (0.3–1.4)	0.28
Educational status of the household head				
	Illiterate	176 (47.7)	Ref	0.52
	Literate	193 (52.3)	1.1 (0.8–1.4)	
Educational status of SAC mother/caretaker				
	Illiterate	252 (68.3)	Ref	0.78
	Literate	117 (31.7)	1.0 (0.7–1.3)	
The education level of SAC mother is conditional on she is literate				
	≤ grade 6	56 (50.5)	Ref	0.29
	>grade 6	55 (49.5)	1.3 (0.8–2.1)	
Is there a pregnant woman in the household of the study SAC?				
	No	317 (85.9)	Ref	0.29
	Yes	52 (14.1)	1.2 (0.9–1.7)	
Is there an under 5 years old child in the household?				
	No	115 (31.2)	Ref	0.36
	Yes	254 (68.8)	1.1 (0.9–1.5)	
Baseline bed net ownership	Not owned	304 (82.4)	Ref	0.52
	Owned	65 (17.6)	0.9 (0.7–1.2)	
Current bed net ownership	Not owned	38 (10.3)	Ref	0.18
	Owned	336 (89.7)	1.3 (0.9–2.1)	
Household size to bed net ratio conditional to owning the bed net is adequate for the household size**				
	> 2(no)	265 (80.1)	Ref	0.02
	≤2(yes)	66 (19.9)	0.7 (0.5–0.9)	
Is the study child slept under the bed net at the bassline survey?				
	Not utilized	343 (93.0)	Ref	0.78
	Utilized	26 (7.0)	0.9 (0.6–1.5)	
Is the study child slept under the bed net at the end line survey? **				
	Not utilized	137 (37.1)	Ref	0.02
	Utilized	232 (62.9)	1.4 (1.1–1.8)	
Wealth index of the household in tertiles**				
	Low	114 (30.9)	0.8 (0.5-1.0)	0.08
	Medium	104 (28.2)	0.8 (0.5-1.0)	0.07
	High	151 (40.9)	Ref	
The altitude of the residence in meters above sea level**				
	≤ 1100	161 (43.6)	Ref	
	1100–1250	157 (42.5)	1.0 (0.7–1.5)	0.89
	> 1250	51 (13.8)	0.4 (0.3–0.7)	< 0.01

**Table 6:** Multivariable analysis of reported malaria incidence among SAC in a MPE intervention study in Southern Ethiopia, 2019–2020

Variable	Categories	#(%) reported malaria	AOR(95%CI)	P-value
Intervention group	No MPE	196 (53.1)	Ref	0.56
	MPE	173 (46.9)	0.9 (0.5–1.4)	
Household size to bed net ratio conditional to owning the bed net is adequate for the household size**				
	> 2(no)	265 (80.1)	Ref	< 0.01
	≤2(yes)	66 (19.9)	0.7 (0.5–0.9)	
Is the study child slept under the bed net at the bassline survey?				
	Not utilized	343 (93.0)		
	Utilized	26 (7.0)		
Is the study child slept under the bed net at the end line survey? **				
	Not utilized	137 (37.1)	Ref	0.02
	Utilized	232 (62.9)	1.4 (1.1–1.8)	
Wealth index of the household in tertiles**				
	Low	114 (30.9)	0.7 (0.5–0.96)	0.03
	Medium	104 (28.2)	0.7 (0.5–0.98)	0.04
	High	151 (40.9)	Ref	
The altitude of the residence in meters above sea level**				
	≤ 1100	161 (43.6)	Ref	
	1100–1250	157 (42.5)	1.0 (0.7–1.5)	0.81
	> 1250	51 (13.8)	0.5 (0.3–0.8)	< 0.01

**statistically significant at multivariable analysis

## Discussion

In our study, bed net utilization among children in the intervention arm increased substantially from 7.9–55.1%% due to a bed net distribution and this rise was similar in both arms (p value:0.92). The MPE did not significantly affected both bed net ownership and its utilization. However, bed net utilization by SAC was associated with adequate access to bed net in the household (i.e., household size to bed net ratio ≤ 2) and with a reported malaria attack in the last 12 months. Cumulative incidence of malaria by consequence was also similar, 16.2% and 18.2% in MPE and control groups respectively. Bed net use was affected by an altitude of the residence place, wealth of the household, adequate access to the bed nets and the use of bed net by the study SAC at baseline of the study. Seeking treatment from a health facility as the 1st choice for malaria in the MPE group was 81.5% compared to 71.9% in control group.

Bed net utilization is one of the effective malaria prevention measures in SSA. SAC who slept under a bed net the previous night before data collection are 22% less likely to be infected by malaria in SSA [51]. At baseline of the study, none of children who slept under the bed net the night before the survey were positive for malaria diagnosed by rapid diagnostic testing [32]. A qualitative evaluation of the MPE revealed that parents in the intervention group had low perceived self-efficacy. In consequence, they need such interventions to be repeated with embedded monitoring to increase adherence to MPE interventions [52]. Teachers-lead participatory education and bed net distribution through a school compound led to significant change in the bed net utilization in the intervention schools as compared to the control schools in Mali [44]. Similar intervention in Iran revealed that regular use of bed net by adults in a household [53], a quasi-experimental study conducted among pregnant women in remote district in Pakistan [54] and pregnant females in the teaching hospital of Osun state, south-west Nigeria [55] have shown that malaria prevention education significantly increased the bed net utilization. The insignificant effect in the current study might be related to providing the MPE one times, which was not the case in other studies.

Apart from the MPE, bed net utilization among SAC was affected by using bed net at bassline survey, household size to bed net ratio equal or less than 2 and reported that the children suffered from malaria. It was also observed in Dar es Salaam [56] and Ghana [57] that people perceived an increased risk of malaria after a malaria attack and as a consequence start using bed nets. It is obvious that in the presence of interest to use bed nets, the bed nets should be available for use by all household members. In such situations those given the highest priority from the household members use the bed net and the rest sleep outside the bed net. In our previous research also bed net utilization was significantly associated with access to adequate bed nets in the households [35]. This indicates that there would be a reduced durability of the bed nets and studying factors affecting the durability of bed nets and adequate access to the household members should be evaluated either to determine the frequency of the bed net distribution campaign or to increase the durability of the bed nets. The association between bed net utilization at baseline and at the end of the intervention could be linked to difference in the background malaria health literacy. In South Sudan, understanding the usefulness of the bed net increased the use of bed nets by the residents [58].

The MPE given by science teachers in the current study did not show significant effect on the cumulative incidence of reported malaria. This reported cumulative incidence of malaria could be overestimated and which may vary between intervention arms. Thus, future studies would consider ecological studies or analysis at cluster level to see the effect of such interventions on laboratory confirmed malaria. Unlike the finding of this study, teachers- lead participatory education given to schoolchildren in Mali significantly affected the point prevalence of malaria in the intervention arm as compared to those in the control arm [44]. Participatory malaria prevention education delivered to the schoolchildren by trained teachers; schoolchildren being involved in the dissemination of malaria message to their community significantly prevented the increase in the prevalence of malaria in the intervention schools which was not the case in the control groups [45]. The difference between the current study and the study in Mali could be related to difference in the background prevalence of malaria and distribution of bed nets in the school along with the education intervention that ensured access to the bed nets to all children.

The percentage of children who sought treatment from a health facility as the 1st choice was not different between arms. The low number of parents attended MPE, health facility problem and poverty could be the reason for the insignificant effect of the intervention on treatment seeking from a health facility. MPE implementation research should be conducted after ensuring the problems hampering seeking treatment from a health facility are removed. In Indonesia, poor understanding of seeking medical care from a health facility was associated with economy and educational status [59]. Qualitative evaluation of the implementation of the current MPE trail revealed that people from the rural area had low perceived self-efficacy. As a result, they requested for repeated MPE intervention to be given with consistent follow-up. In Rwanda, having a knowledge of malaria symptoms affected the prompt diagnosis and getting care from a health facility [60]. School based malaria management could be an option to solve economic problem that hinders access to treatment from a health facility as was done in Malawi [61, 62].

One of the limitations of our study was the MPE being given only once, a year before the evaluation of its effectiveness. This might be presumptive reason for the insignificant effect of the intervention on the outcome variables considered. The 2nd limitation of the study was failure to include SAC not enrolled to a school. These children may differ in certain aspects affecting the use of malaria prevention measures. The third limitation was low number of parents attended the MPE. The other limitation was that bed net coverage was very low in the study area at the baseline of the study which might underestimate the true effect on the reported cumulative incidence of malaria. The strength of this study was that it is a large study that involved children from both urban and rural areas of hard-to reach settings.

## Conclusions

The bed net distribution in the study area substantially increased both the ownership and bed net utilization by SAC. MPE given one time to the SAC and their parents has no significant effect on the bed net utilization, treatment seeking from a health facility and cumulative reported incidence of malaria. Sustainable implementation efforts have to be ensured before organizing any MPE program especially for SAC, a neglected but relevant, vulnerable and major reservoir. The bed net utilization by SAC was associated with the household size to the bed net ratio, bed net utilization at baseline and reported attack by malaria in the last 12 months. Reported incidence of malaria was associated with an altitude of residence place, wealth index, adequate access to the bed net and the bed net use by SAC. School-based bed net distribution and management of malaria could be an option to ensure access to malaria prevention measures.

## Data Availability

The datasets used and/or analyzed during the current study are available from the corresponding author on reasonable request.

## Abbreviations

ACT: artemisinin-based combination therapy
AIC: Akaike Information Criterion
AOR: Adjusted Odds Ratio
CI: confidence interval
COR: Crude odds ratio
COVID-19: Coronavirus Disease 2019
CSV: comma-separated value
DHS: Demographic and Health Surveys
GPS: global positioning system
ICC: intracluster correlation coefficient
IRB: Institutional Research Ethics Review Board
IRB: Research Ethics Review Board
ITN: Insecticide-treated bed net
LLINs: long-lasting insecticide-treated nets
MASL: Meters above sea level
MPE: Malaria prevention education
ODK: Open Data Kit
PAR: Participant
PDAT: Prompt diagnosis and treatment
SAC: School-aged children
SNNPR: Southern Nations, Nationalities, and Peoples Regional
SSA: sub-Saharan Africa
WHO: World Health organization
